# OPPORTUNITIES FOR INTEGRATING A GSA STUDENT CHAPTER WITH A SIGMA PHI OMEGA HONOR SOCIETY CHAPTER

**DOI:** 10.1093/geroni/igae098.1323

**Published:** 2024-12-31

**Authors:** Katarina Friberg-Felsted

**Affiliations:** University of Utah, Salt Lake City, Utah, United States

## Abstract

The University of Utah Gerontology Interdisciplinary Program (GIP) has had an active Sigma Phi Omega chapter, Alpha Chi, for decades. Students are enrolled in one of our five programs: 1) Master of Science in Gerontology (MSG), 2) MSG/ Master in Physician Assistant Studies (MPAS) dual degree, 3) Graduate Certificate in Gerontology, 4) Undergraduate Certificate in Gerontology, and 5) Minor in Gerontology. Students are invited to join Alpha Chi in the second semester of their program. Alpha Chi’s Executive Committee ideally contains a student President, a student Secretary, and a student Treasurer; sometimes these positions are held by one or two students. The Alpha Chi Executive Committee is supported by a faculty advisor, who holds the position for several years. To integrate a GSA Student Chapter with our SPO local chapter, we began by following the guidelines in the GSA Student Chapter Handbook. Our process included application for a student group recognition on campus, verifying that our current Executive Committee members were student members of GSA, spreading the word through a variety of pathways, and ensuring that our events and projects were co-sponsored by Alpha Chi Chapter and GSA Student Chapter. This presentation will discuss both process and outcomes of the integration, including more students being involved earlier and more students being introduced to GSA student benefits, including ESPOs offerings.

